# Violence against women: where are we 25 years after ICPD and where do we need to go?

**DOI:** 10.1080/26410397.2019.1676533

**Published:** 2019-11-08

**Authors:** Claudia Garcia-Moreno, Avni Amin

**Affiliations:** a Medical Officer, Team Lead Violence against Women, Department of Reproductive Health and Research/Human Reproduction Programme, World Health Organization, Geneva, Switzerland. *Correspondence*: garciamorenoc@who.int; b Technical Officer-Violence against Women, Department of Reproductive Health and Research/Human Reproduction Programme, World Health Organization, Geneva, Switzerland

## Background

In 1994, the Programme of Action of the International Conference on Population and Development (ICPD) in Cairo noted that gender equality and equity, empowerment, and elimination of violence against women (VAW) were cornerstones of development.^1^ The Beijing Platform for Action followed, urging actions to prevent and eliminate VAW, including through legal reform, research and data collection.^2^ The gains made in putting VAW on the global development agenda in the 1990s were the result of strategic, intentional and thoughtful advocacy and work by many women’s organisations with support from some governments. The last 25 years have seen progress in addressing VAW in three ways: the recognition that, as well as a social, development and human rights issue, VAW is an urgent public health problem; the availability of both prevalence data and increased evidence on what works to prevent and respond to VAW; and more governments putting in place laws and policies and implementing programmes to address VAW.

### Recognition of the problem

It was not until 2015 that long-standing advocacy culminated in the inclusion of a specific target (5.2) and indicators on “the elimination of all forms of VAW and girls” in the Sustainable Development Goal 5 on promoting gender equality and empowerment of women. The inclusion of a VAW target – which had not been possible in the Millennium Development Goals in 2000 – was made possible by the growing availability of data and the evidence-based advocacy of women’s organisations, UN and other organisations. Regionally, inter-governmental commitments were also adopted, e.g. the 1994 Inter-American (or Belem do Para) Convention, the 2011 Council of Europe Istanbul Convention and the 2003 Maputo Protocol in Africa.

In 2016, the World Health Assembly, a gathering of ministers of health from 193 governments, endorsed a global plan of action to strengthen the role of the health system in addressing violence, in particular against women and children.^3^ This recognised the need for urgent action to address VAW and highlighted the responsibilities of governments. Continuous evidence-based advocacy, alongside WHO guidelines and implementation tools on the health sector response to intimate partner violence (IPV) and sexual violence, is contributing to the gradual integration of strategies to address VAW and girls in existing health programmes like those for HIV prevention, adolescent health, sexual and reproductive health including maternal health, and mental health. Challenges remain in the integration of VAW efforts in the health system in different country contexts such as: lack of inclusion of violence-response in health professionals’ curricula, no clear policies on partner violence, and lack of coordination among various actors and departments involved in planning and delivering integrated services.

### Availability of data and research

The Beijing Platform of Action (para 120) noted that “The absence of adequate gender-disaggregated data and statistics on the incidence of violence makes the elaboration of programmes and monitoring of changes difficult”.^2^ The lack of agreed measures of different forms of violence that could be used across cultures limited the availability and quality of data available, particularly from low- and middle-income countries. This was addressed with the WHO multi-country study on women’s health and domestic violence and the addition of a module on domestic violence in the Demographic and Health Surveys,^4,5^ with more countries now collecting prevalence data on different forms of VAW. In 2013 WHO and partners published the first global and regional estimates for IPV and non-partner sexual VAW^6^ based on IPV prevalence data from 79 countries and two territories. The database now includes data from 153 countries. The availability of data on non-partner sexual violence also increased from 52 to 92 countries, however quality is poor and measurement and reporting of sexual violence need strengthening. The same report^6^ documented some health impacts of violence, particularly on the mental health and sexual and reproductive health of women including increased odds of unintended pregnancies, induced abortions, sexually transmitted infections, depression and substance use. Violence and fear of violence can be barriers to contraceptive use and reproductive coercion is recognised as part of control and abuse, although data on this are scarce. Availability of prevalence data and its health consequences has been instrumental in getting governments to recognise VAW as a public health problem and to take action, e.g. by adopting a law, national policy, plan of action or programme.

The last 5–10 years have seen a growth in funding for research to evaluate interventions. The UK Development Fund for International Development (DFID) alone dedicated £25 million to *What Works to Prevent VAW and Girls*, a five-year research initiative that has already identified some promising or effective interventions. WHO, with UN Women, has produced *RESPECT women: A framework for preventing VAW*, which summarises evidence on promising programmes under seven strategies (**R**elationships strengthened, **E**mpowerment of women, **S**ervices ensured, **P**overty reduced, **C**hildhood abuse reduced/eliminated, **T**ransformed norms, attitudes and behaviours). Gaps remain, but we are learning that successful programming strategies focus on women’s safety, address unequal gender power relations, apply participatory approaches that stimulate critical reflections about power, strengthen the agency of communities and facilitate partnerships. Comprehensive prevention programmes are still lacking.

### Government action

The number of countries addressing VAW in their laws and policies has increased since ICPD. Of 141 countries in 2017, 76% have laws on domestic violence.^7^ Only 42% of 189 countries have legislation that explicitly criminalises marital rape,^8^ 40% have provisions that cover sexual harassment in education and 18% that cover sexual harassment in public places.^7^ Nearly 40% of countries (out of 187 for which data are available) have laws that discriminate against women’s property rights, which can impede women leaving abusive relationships.^9^ Many countries also have national or subnational plans of action to address VAW and are implementing prevention programmes. The implementation of laws, policies and programmes remains weak.

Evidence suggests that secondary education, women’s employment, access to economic resources and inheritance rights protect against violence.^10^ These are markers of women’s empowerment, but can potentially enable women to leave abusive relationships by reducing economic dependency on their partners. Women’s and girls’ access to education has improved, although in Sub-Saharan Africa, North Africa and West Asia, girls continue to be disadvantaged in completing secondary education.^11^

Few countries are implementing programmes at scale. This is due to lack of political will, competing priorities and lack of government accountability in addressing gender equality and women’s empowerment. Hardly any are allocating significant resources from domestic budgets and VAW programming is a small percentage of overseas development assistance. In 2018, only 0.12% of the total allocation to humanitarian funding was allocated to addressing VAW.^12^ Governments need to demonstrate political will, allocate resources for the reduction and eventual elimination of VAW, and ensure enabling conditions (e.g. legal frameworks that do not discriminate against women and girls, policies that support gender equality including parental leave and childcare, access to secondary education, engaging with women’s organisations).

## The way forward

Progress has been made and, going forward, we must continue to build on national and global feminist movements like #Metoo, #Ni una mas (not one more) and many others, increase investments in women’s rights organisations as the backbone of advancing this agenda, and hold governments accountable. Second, while continuing to support data and research to identify what works, we must expand our understanding of the multiple forms of sexual abuse and harassment that women suffer, including in the workplace and schools, as well as of the new forms of abuse such as through mobile phones, internet and social media. Third, we must significantly invest domestic and international resources in the implementation of the laws, policies and plans of actions to scale-up promising and effective prevention and response interventions, and transform norms related to masculinity that are premised on the exercise of power over women, and end the acceptability of VAW and girls. We also need to integrate VAW responses in undergraduate and in-service training of legal, judicial and health personnel to support women affected by violence to achieve their full potential. In the next 25 years we must strive and can achieve a world where all women and girls, everywhere are free of discrimination, violence and coercion.

## References

[1] UN Population Fund (UNFPA) Report of the International Conference on Population and Development; 1994 Sep 5–13; Cairo.A/CONF.171/13/Rev.1; 1995. [cited 2019 Aug 19]. http://www.refworld.org/docid/4a54bc080.html.

[2] United Nations Beijing Declaration and Platform of Action, adopted at the Fourth World Conference on Women; 1995 Oct 27. [cited 2019 Aug 19]. http://www.refworld.org/docid/3dde04324.html.

[3] Global plan of action to strengthen the role of the health system within a national multisectoral response to address interpersonal violence, in particular against women and girls, and against children. Geneva: WHO; 2016.

[4] WHO multi-country study on women’s health and domestic violence against women: summary report of initial results on prevalence, health outcomes and women’s responses. Geneva: World Health Organization; 2005. [cited 2019 Aug 19]. https://apps.who.int/iris/bitstream/handle/10665/43310/9241593512_eng.pdf;jsessionid=9434D0F18E7480464290CA96347FA5D6?sequence=1.

[5] Domestic violence module: demographic and health surveys methodology. Washington (DC): United States Agency for International Development; 2014. [cited 2019 Aug 19]. https://dhsprogram.com/pubs/pdf/DHSQMP/DHS6_Module_Domestic_Violence_6Aug2014_DHSQMP.pdf.

[6] WHO, LSHTM, SAMRC Global and regional estimates of violence against women: prevalence and health burden of intimate partner violence and non-partner sexual violence. Geneva: WHO; 2013.

[7] Tavares P, Wodon Q. Global and regional trends in women’s legal protection against domestic violence and sexual harassment. Ending violence against women notes series Washington (DC): World Bank; 2017 [cited 2019 Aug 19]. http://pubdocs.worldbank.org/en/679221517425064052/EndingViolenceAgainstWomenandGirls-GBVLaws-Feb2018.pdf.

[8] Progress of the world’s women: 2019–2020. New York: UN Women; 2019. [cited 2019 Aug 19]. https://reliefweb.int/sites/reliefweb.int/files/resources/Progress-of-the-worlds-women-2019-2020-en.pdf.

[9] The women, business and law database. Washington (DC): World Bank; 2018. [cited 2019 Aug 19]. https://wbl.worldbank.org/en/data/exploretopics/managing-assets.

[10] Heise L, Kotsadam A. Cross-national and multilevel correlates of partner violence: an analysis of data from population-based surveys. Lancet Global Health. 2015;3(6):E332–E340. doi: 10.1016/S2214-109X(15)00013-3 26001577

[11] UNESCO Global education monitoring report: gender report: building bridges for gender equality. Paris: UNESCO; 2019 [cited 2019 Aug 19]. https://unesdoc.unesco.org/ark:/48223/pf0000368753/PDF/368753eng.pdf.multi.

[12] VOICE and IRC Where is the money? How the humanitarian system is failing in its commitments to end violence against women and girls. New York: IRC; 2019 [cited 2019 Aug 19]. https://reliefweb.int/sites/reliefweb.int/files/resources/whereisthemoneyfinal.pdf.

